# Primary Healthcare Leaders’ Perceptions of Relational Leadership: An Action Learning Research Approach

**DOI:** 10.1155/jonm/9979506

**Published:** 2026-10-28

**Authors:** Lillian Kalimashe, Emmerentia du Plessis, Leepile Alfred Sehularo

**Affiliations:** ^1^ Department of Health Faculty of Health Sciences West Rand District Health Services Gauteng Department of Health Johannesburg South Africa; ^2^ NuMIQ Research Focus Area Faculty of Health Sciences North-West University Potchefstroom South Africa nwu.ac.za; ^3^ Lifestyle Diseases Research Focus Area Faculty of Health Sciences North-West University Mafikeng South Africa nwu.ac.za

**Keywords:** action learning group, leadership, primary healthcare, relational leadership

## Abstract

**Background:**

In complex healthcare settings, such as primary healthcare, effective leadership is critical for fostering an environment that prioritizes patient‐centered care and strengthened team functioning. Effective leadership requires approaches that emphasize meaningful relationships, collaboration, and communication. It is different from conventional leadership, which deals with delegating authority and tasks. Little is known about how PHC leaders in under‐resourced South African settings perceive and enact relational leadership (RL) in their daily practice.

**Aim:**

This study explored PHC leaders’ perceptions of RL within a South African primary healthcare setting in Gauteng Province, using a qualitative action learning research approach.

**Research Design:**

A qualitative action learning research approach was used in this study. The data were collected from April 2024 to September 2024.

**Participants and Research Context:**

Ten leaders participated in monthly action learning group sessions, reflective journaling, and collaborative discussions over 6 months, during which the primary researcher observed interactions. Data from transcripts, journals, and the observational notes were thematically analyzed.

**Ethical Considerations:**

Ethical approval was obtained from the North‐West University Health Research Ethics Committee (NWU‐00041‐23‐A1). All participants signed a written informed consent form before forming part of the action learning group.

**Results:**

Five themes emerged: understanding and implementing RL; benefits of RL; challenges while implementing RL; connection between RL and patient care; and strategies to enhance the practice and implementation of RL.

**Conclusions:**

Participants perceived RL as fostering trust, team cohesion, and open communication, and as supporting patient‐centered care, although heavy workloads, limited resources, and hierarchical norms constrained its practice. These reflections represent subjective perceptions rather than objectively measured outcomes, but they highlight RL’s potential value for leadership development in PHC settings.

## 1. Background

Strengthening leadership in primary healthcare (PHC) is imperative [1], particularly in settings where patient‐centered care, teamwork, and service coordination are essential for quality outcomes. Relational leadership (RL) has gained increasing attention as a leadership approach that emphasizes meaningful relationships, collaboration, communication, ethical behavior, and inclusiveness. Cleary et al. [2] highlight the importance of building an evidence base for RL in health systems, especially in low‐ and middle‐income countries, arguing that understanding how RL works and how it can be developed is crucial for strengthening health system performance.

The context of this study is the West Rand Health District (WRHD) in Gauteng Province, South Africa, a region facing complex public health challenges, including resource constraints, communication gaps, and fragmented teamwork. These contextual realities underscore the need for leadership approaches that promote connection, shared purpose, and collaborative problem‐solving.

Research indicates that RL stands out from conventional leadership approaches because it prioritizes relationships, team cohesion, and collective engagement as central tenets of leadership [3, 4]. Relational leaders engage in dialog to make sense of complex issues, solve problems, and shape strategic directions that support collaborative action [5]. Caring, empathy, and ethical engagement are also highlighted as essential attributes of relational leaders [1, 6]. In healthcare settings, RL is recommended because it fosters interprofessional communication, teamwork, and alignment of departmental functions, all of which are critical for addressing the complex challenges faced in PHC environments [7, 8].

Healthcare systems are dynamic and constantly evolving, requiring leadership approaches that are proactive, inclusive, and supportive of staff well‐being. Figueroa et al. [9] describe health systems as “sophisticated and constantly changing across diverse settings and health service levels,” reinforcing the need for leadership models that can meaningfully guide, support, and empower teams. RL is therefore particularly relevant in PHC settings, where leaders must navigate complexity while promoting cohesion, service quality, and patient‐centered care [2, 7].

Despite these documented benefits and that RL has been explored in various healthcare contexts, little is known about how PHC leaders in under‐resourced South African settings perceive and enact RL in their daily practice [10]. Existing studies [2, 4, 7] highlight RL’s importance for collaboration and communication, yet few have examined leaders’ lived experiences, the contextual barriers they face, or how RL develops through reflective practice.

This study addresses these gaps by exploring WRHD healthcare leaders’ perceptions of RL through an action learning approach. By capturing real‐time reflections, collaborative discussions, and the primary researcher’s observations, the study provides a distinctive account of how leaders interpreted and applied RL principles while navigating the daily realities of a PHC environment facing multiple challenges.

## 2. Materials and Methods

The comprehensive reporting approach taken for the following materials and methods sections aligns with the COREQ criteria, which ensure rigor and depth during the research process.

### 2.1. Design

This study employed a qualitative action learning research design, which was well suited to exploring leaders’ perceptions of RL within a real‐world PHC context. Action learning is grounded in solving actual organizational challenges while fostering continuous, on‐the‐job learning through reflection, dialog, and collaborative problem‐solving [11]. This approach aligned directly with the study’s aim, as it enabled PHC leaders to examine their leadership practices, test RL principles, and reflect on their experiences in real time [11].

The action learning process comprised an initial workshop designed to strengthen participants’ understanding of RL concepts, followed by six monthly action learning group sessions. Each session lasted approximately 1 h and created a structured yet flexible environment in which leaders could critically evaluate their leadership behaviors in relation to RL principles. The interactive nature of these sessions encouraged continuous reflection, adaptation, and the practical application of RL in everyday leadership situations. This design therefore generated rich, practice‐based insights into how RL can be implemented and how it influences leadership behavior in PHC settings.

Relational leadership theory (RLT) [12] provided the conceptual foundation for the study. RLT views leadership as a social process that emerges through interactions among individuals, emphasizing mutual respect, ethical engagement, inclusiveness, and collaborative sense‐making. This theoretical lens was particularly relevant in the under‐resourced PHC context explored in this study, where leaders often navigate complexity not through authority, but through human connection, adaptability, and shared problem‐solving. RLT therefore offered an ideal framework for examining how leaders interpret and enact RL principles in their daily practice.

The integration of action learning with RLT strengthened the methodological approach by enabling leaders to reflect on their experiences, learn from them, and continuously refine their RL practices. This combination was crucial for addressing the dynamic challenges of PHC environments and for generating a deeper understanding of how RL can enhance teamwork, communication, and service delivery. Overall, the design provided a comprehensive and contextually relevant approach to exploring PHC leaders’ perceptions of RL and its practical application in a complex healthcare setting.

### 2.2. Sample

The study included 10 healthcare leaders from a health district in Gauteng Province, South Africa, who participated in monthly action learning group sessions over 6 months. A fixed cohort of 10 while still retaining variation across leadership roles was selected because action learning groups function optimally with small, interactive cohorts that allow for deep reflection, mutual learning, and sustained engagement. Although the sample size was determined based on the practical requirements of action learning group facilitation, data saturation was also considered throughout the study. Saturation was approached when no new codes or insights emerged during the final action learning sessions, reflective journal entries, or observational notes. By the fifth and sixth sessions, participants’ reflections consistently reinforced previously identified patterns, indicating that the core concepts and experiences related to RL had been sufficiently captured. The convergence of themes across group discussions, journals, and observations further supported that saturation had been achieved within this sample.

Participants were purposively recruited using inclusion and exclusion criteria to ensure representation across different leadership positions in the organization [13], and their demographic characteristics were recorded.

To determine the study sites, the district’s four subdistricts served as the sampling frame. A simple random sampling procedure was used to select two of the four subdistricts: each subdistrict was listed, assigned a number, and a computer‐generated random number sequence was used to select two. This approach reduced site‐selection bias and ensured that the chosen subdistricts were not influenced by researcher preference or contextual familiarity. Only these two subdistricts were included because the action learning process required repeated engagement over 6 months, and expanding the study to all four subdistricts would have exceeded logistical and time constraints. The selected subdistricts provided sufficient diversity in leadership roles, facility types, and operational challenges to support meaningful exploration of RL perceptions.

Only leaders working within the two randomly selected subdistricts were eligible for inclusion because the repeated sessions and reflective activities required sustained participation within a manageable geographical area. The inclusion criteria were holding an officially appointed leadership position, working within either selected subdistrict, and willingness to participate. Leaders in acting positions were excluded because temporary appointments might not last for the duration of the study and may confer limited decision‐making authority. Leaders outside the selected subdistricts and those unwilling to participate were also excluded.

The final sample included clinical, administrative, and front‐line supervisors from different leadership levels. This diversity enabled exploration of varied experiences of applying RL in PHC and supported a comprehensive understanding of how RL is interpreted and enacted across leadership contexts.

### 2.3. Data Collection

Data collection occurred between April and September 2024 and drew on three sources: transcriptions of the monthly action learning group sessions, participants’ reflective journal entries, and the primary researcher’s observational notes. Collecting data over a prolonged period and from multiple sources enhanced depth, triangulation, and contextual richness.

Each action learning group session lasted approximately 1 h and focused on discussions related to RL. To accommodate the demanding schedules of healthcare leaders, participants were offered a choice between face‐to‐face and virtual sessions; consistent with previous research highlighting the convenience of virtual engagement [14], participants elected to meet online. All online sessions were audio‐recorded and transcribed verbatim.

#### 2.3.1. Action Learning Group Discussions

During the monthly sessions, participants discussed their leadership experiences, challenges, and attempts to apply RL principles in their daily practice [15]. They shared concerns, strategies, and reflections, and received peer feedback on their use of RL. These discussions fostered an atmosphere of knowledge sharing, mutual support, and collaborative problem‐solving, which are key features of the action learning approach.

However, the group‐based nature of these discussions introduced the possibility of social desirability and group dynamics influencing participants’ responses. To mitigate these influences, the facilitation model, reflexive practices, and triangulation strategies were intentionally designed to support authentic engagement and reduce undue conformity. These safeguards are described below.

#### 2.3.2. Role and Expertise of the Facilitator

The action learning groups were co‐facilitated by two individuals: a healthcare leader from the participant cohort, serving as the primary facilitator, and the primary researcher, serving as co‐facilitator. The facilitator was selected based on voluntary interest, ensuring participant‐led ownership and contextual authenticity. Their responsibilities included planning sessions, maintaining focus, and fostering a psychologically safe environment where participants felt comfortable sharing experiences and challenges.

The co‐facilitator, experienced in qualitative inquiry, action learning, and RL, guided discussions to remain aligned with RL concepts and emerging issues. They documented observations which included nonverbal cues, emotional tone, and interaction patterns to enhance data quality. Recognizing the potential influence of this dual role, steps were taken to reduce power dynamics: shared facilitation responsibilities, transparent role boundaries, and encouragement of participant‐led discourse. The co‐facilitator also engaged in reflexive journaling throughout the process to interrogate assumptions, monitor potential bias, and uphold ethical engagement.

To further enhance validity and minimize interpretive bias, peer debriefing with an independent co‐coder was conducted during analysis, and member checking with two participants was undertaken to confirm emergent themes. This dual‐facilitation model upheld the relational ethos of the study and empowered participants as co‐constructors of knowledge.

#### 2.3.3. Reflective Journals

Participants were encouraged to maintain reflective journals throughout the data collection period. They documented their thoughts, experiences, and insights related to RL, guided by prompts that supported structured reflection. Participants were asked to write at least weekly and ideally after each action learning session to capture immediate reactions. These reflections enabled participants to critically evaluate their leadership challenges, strategies used, and perceived outcomes, thereby deepening their engagement with RL concepts.

#### 2.3.4. Researcher’s Observation Notes

The primary researcher recorded detailed observation notes during each session, capturing the dynamic exchanges among participants. These notes included descriptions of group interaction patterns, turn‐taking, flow of discussion, and how ideas were questioned or expanded. Nonverbal cues such as nods, gestures, facial expressions, and emotional tone were documented to contextualize participants’ verbal contributions and enhance interpretive depth.

The researcher also noted moments of excitement, frustration, clarity, and collective problem‐solving, as well as instances where participants supported one another by sharing similar experiences or offering advice. Reflexive notes were kept throughout, capturing the researcher’s impressions, emerging patterns, and evolving themes. These multilayered observations enriched the dataset and strengthened the researcher’s ability to interpret participants’ perceptions of RL within the PHC context.

### 2.4. Data Analysis

Inductive thematic analysis was used to identify patterned meaning across the data [16]. Analysis began with repeated reading of action learning group transcripts, reflective journals, and observational notes to support familiarization [16]. The three sources were treated as an integrated data set while retaining source labels so that similarities and differences could be traced. The first author generated initial, primarily descriptive codes from segments relevant to the research aim. Codes were compared across participants, sessions, and sources, merged when they captured the same idea, separated when they contained distinct meanings, and organized into provisional categories. The research team then examined relationships among categories, developed candidate themes and subthemes, and checked each candidate against the coded extracts and the full data set. Theme names and definitions were revised iteratively to improve internal coherence, distinction between themes, and alignment with the study aim.

An independent co‐coder reviewed the data and developed a parallel set of codes. The first author and co‐coder compared code meanings and boundaries, discussed differences, and reached agreement through deliberation rather than relying on a numerical reliability threshold [17]. They then refined the thematic structure and documented key analytic decisions in the audit trail. Triangulation across group discussions, journals, and observational notes was used to examine convergence and divergence, while member checking with two participants assessed whether the resulting themes were recognizable representations of participants’ accounts. This systematic and iterative approach ensured that the final themes were grounded in the data and reflected a trustworthy interpretation of participants’ perspectives.

### 2.5. Rigor of the Study

Rigor was ensured through credibility, dependability, confirmability, and transferability strategies [18, 19]. Credibility was supported through prolonged engagement, triangulation of data sources, co‐coding, and member checking. Dependability was strengthened through detailed documentation of coding iterations and analytic decisions. Confirmability was enhanced by maintaining an audit trail that recorded coding processes, theme refinement, and methodological decisions. Transferability was supported by providing thick descriptions of the setting, participants, and context.

### 2.6. Ethical Considerations

Ethical considerations were paramount throughout this research study. Ethical approval was obtained from the North‐West University Health Research Ethics Committee (NWU‐00041‐23‐A1), ensuring the research adhered to established ethical standards. Approval was also obtained from the relevant health authorities to conduct the study in their district. The study was conducted in accordance with the Declaration of Helsinki. Each participant completed a consent form before participating in the study [20]. The participants were informed of the study’s aim, approach, and expectations. They were further made aware of their right to discontinue their participation in the study at any time without explanation. While participants were not anonymous to the researchers or fellow participants, the data were anonymized to ensure their privacy and confidentiality. Group rules for confidentiality were established, requiring all participants to maintain the privacy of shared information. The research team signed letters of commitment to confidentiality, and data were safeguarded, barring any unauthorized attempt at acquisition.

## 3. Results

### 3.1. Demographic Characteristics of Participants

All 10 participants were in official healthcare leadership positions and thus met the inclusion criteria. Eight participants were female, two were male, and each had a diploma or degree. All participants managed a significant number of staff. The age range of the participants was between 33 and 52 years, with a mean age of 41.8 years. Each participant attended at least 5 of the 6 action learning group sessions. Table 1 presents the general demographics of the participants.

**TABLE 1:** Demographics of participants (*n* = 10).

Participant no.	Gender (M/F)	Age	Education	Leadership title
1	F	37	Diploma	Facility Manager
2	F	52	Diploma	Coordinator
3	F	44	Degree	Coordinator
4	F	51	Degree	Assistant Manager
5	F	38	Degree	Facility Manager
6	F	37	Diploma	Facility Manager
7	M	33	Degree	Coordinator
8	M	35	Degree	Admin Manager
9	F	41	Diploma	Monitoring & Evaluation Manager
10	F	50	Diploma	Facility Manager

### 3.2. Thematic Findings

The story of the health district and its subdistricts reflects how participants perceived their leadership experiences during the action learning process. While many leaders described shifts toward more collaborative, communicative, and relational practices, these accounts represent subjective perceptions rather than objectively verified improvements. The themes therefore illustrate how participants experienced and interpreted RL within the intervention context, rather than demonstrating measurable changes in team dynamics or patient care. For example, when a participant noted that “Being approachable has transformed how my team interacts with me” (ALG 4, Participant 7), this reflects a personal impression of change rather than an independently assessed outcome. Similarly, reflections on enhanced teamwork or improved patient experiences should be understood as participants’ interpretations of their leadership practice during the study period. As such, the findings should be read as insights into leaders’ perceived RL experiences, not as evidence of organizational or clinical impact.

#### 3.2.1. Reflections From the Participants

“One major change has been a shift towards more frequent and intentional communication with my team members. I have made a conscious effort to check in regularly with everyone, both to provide support and guidance” (action learning Group 4, Participant 1).

“Initially I was not so sure whether ‘relational leadership’ is practical because as a leader that is above 50 years, I believed leadership was based on hierarchy and giving instructions. I now feel confident that I can build relations based on caring and compassion with my team and still be an effective leader that gets goals achieved” (action learning Group 6, Participant 2).

“I loved the fact that one had to practice relational leadership on the job and see what best fits the context, classroom‐based leadership training tends to not recognize context as a factor that determines the effectiveness or ineffectiveness of the leadership development training. With the use of relational leadership, I am now able to apply what I have learned to the real work environment” (reflective journal entry, Participant 5).

#### 3.2.2. Perceptions of RL Among PHC Leaders

The five themes generated in this study include “understanding and implementing relational leadership”, “benefits of relational leadership”, “challenges while implementing relational leadership”, “connection between relational leadership and patient care”, and “strategies to enhance the practice and implementation of relational leadership”. In total, 22 subthemes were generated from these 5 themes (see Table 2 for summary of themes and subthemes). In the next section, the themes and subthemes are presented, supported by quotes from the data set. The quotes are contextualized using the following acronyms: ALG = action learning group, RJE = reflective journal entry, ON = observational note.

**TABLE 2:** Summary of themes and subthemes.

Themes	Subthemes
Understanding and implementing relational leadership	• Shift from traditional leadership to a relational approach • Self‐awareness, introspection, and continuous learning • Shared decision‐making • Open communication and approachability

Benefits of relational leadership	• Improved team dynamics and stronger relationships • Collective growth, self‐development, and empowerment • Enhanced accountability • Innovation and adaptability • Constructive conflict resolution

Challenges while implementing relational leadership	• Limited resources and support • Systemic barriers • Heavy workload • Resistance to change • Balancing care and accountability

Connection between relational leadership and patient care	• Role‐modeling of patient‐centered care • Enhanced communication and collaboration in healthcare teams • Adaptation to changing healthcare contexts

Strategies to enhance the practice and implementation of relational leadership	• Equal opportunities for professional development • Regular feedback‐seeking and self‐reflection • Knowing team members on a personal level • Clear communication of vision and goals • Development of emotional intelligence

##### 3.2.2.1. Understanding and Implementing RL

Participants described how they believed their understanding of RL evolved over time. They reported becoming more deliberate in checking in with staff, listening actively, and creating opportunities for open dialog. The action learning process helped participants reflect on their leadership styles and begin integrating relational behaviors into routine practice. Several leaders noted that RL required conscious effort and consistency, especially when shifting from long‐standing hierarchical habits.

Within this theme, participants described the shift from traditional leadership to a relational approach as one that required self‐awareness, introspection, and continuous learning, as they examined how their own behaviors affected their teams. Leaders also described moving towards shared decision‐making by involving staff in discussions rather than relying solely on directives. Open communication and approachability were viewed as central to this shift, with leaders making themselves more available to staff and encouraging them to raise concerns.

“I have made a conscious effort to check in regularly with everyone, both to provide support and guidance” (ALG 6, Participant 1).

These reflections illustrate how leaders began to internalize RL as a practical, everyday leadership stance rather than a theoretical concept.

##### 3.2.2.2. Benefits of RL

Participants perceived several benefits associated with practising RL. Leaders described feeling more connected to their teams and more confident in their ability to support staff. They reported that relational behaviors encouraged openness, reduced tension, and fostered a more supportive work environment. Some participants also noted perceived improvements in patient interactions and workplace atmosphere, although these impressions were subjective and not objectively measured.

Beyond improved team dynamics and stronger relationships, participants associated RL with collective growth, self‐development, and empowerment, as staff were encouraged to contribute ideas and take initiative. Leaders also perceived enhanced accountability, describing staff as taking greater ownership of their responsibilities when they felt included and supported. Some participants linked RL to innovation and adaptability, noting that open dialog made it easier for teams to suggest and try new ways of working. In addition, leaders described more constructive conflict resolution, with disagreements addressed through dialog rather than avoided or escalated.

“Being approachable has transformed how my team interacts with me” (ALG 4, Participant 7).

Overall, RL was viewed as a meaningful approach for strengthening everyday leadership practice and fostering supportive team environments.

##### 3.2.2.3. Challenges While Implementing RL

Despite the perceived benefits, participants identified several challenges in applying RL. Leaders described how organizational pressures and resource limitations sometimes hindered their ability to practice RL consistently. Some felt that relational approaches required more time and emotional investment than traditional leadership styles. Others noted that staff accustomed to directive leadership sometimes struggled to adjust to more collaborative approaches and became resistant.

Participants further pointed to systemic barriers, such as entrenched hierarchical structures and bureaucratic processes, which limited their discretion to lead relationally. Leaders also described the difficulty of balancing care and accountability: they sought to remain supportive and compassionate towards staff while still addressing performance concerns and ensuring that service expectations were met.

“Sometimes the workload makes it hard to practice relational leadership consistently” (Reflective journal).

These challenges highlight the contextual realities of PHC settings and the need for supportive structures to sustain relational practices.

##### 3.2.2.4. Connection Between RL and Patient Care

Participants perceived a link between RL and patient care quality. Leaders felt that improved team communication and emotional support contributed to more coordinated care and more positive patient experiences. While these reflections were subjective, they illustrate how leaders made sense of RL’s potential influence on service delivery, even though objective indicators were not collected.

“Ever since I started practicing relational leadership, I started seeing a lot of compliments in our facility’s suggestions’ box” (RJE, Participant 7).

The leaders believed that role modeling relational behaviors such as empathy, active listening, and collaborative problem‐solving helped create a more patient‐centered environment [21].

Participants also linked RL to enhanced communication and collaboration in healthcare teams, which they believed supported better coordination of patient care. In addition, leaders described how relational approaches helped them and their teams adapt to changing healthcare contexts, such as shifting service demands and operational pressures, while maintaining a focus on patients’ needs.

##### 3.2.2.5. Strategies to Enhance the Practice and Implementation of RL

Participants proposed several strategies to strengthen RL in PHC settings, including mentorship, reflective practice, collaborative decision‐making, and structured opportunities for team dialog. Action learning was viewed as a valuable platform for developing relational skills. Leaders emphasized the importance of emotional intelligence (EI), self‐awareness, and emotional regulation competencies. Participants also highlighted the need for organizational support, including leadership development programmes and protected time for reflection.

Participants emphasized that equal opportunities for professional development were important so that all staff, a few, could grow and contribute. Regular feedback‐seeking and self‐reflection were described as practical ways for leaders to monitor and adjust their own behavior. Leaders also stressed the value of knowing team members on a personal level, which they felt helped them understand staff needs and respond with empathy. Clear communication of vision and goals was seen as essential for aligning relational practice with service expectations, while the development of EI was viewed as underpinning these strategies.

“Reflecting after each session helped me see where I needed to improve” (RJE, Participant 4).

The last theme captures the different approaches participants reported helped them to improve their practice and implementation of RL.

These five themes collectively illustrate how PHC leaders interpreted, applied, and experienced RL within their daily practice. They also highlight the contextual realities that shape leadership behaviors in under‐resourced PHC settings. The Discussion section explores these findings in relation to existing literature and considers their implications for nursing management practice.

## 4. Discussion

This qualitative study explored PHC leaders’ perceptions of RL within an action learning context. The findings demonstrate how leaders made sense of RL in their daily practice and how reflective engagement supported a gradual shift from hierarchical, authority‐driven approaches toward more relational, collaborative, and communicative leadership behaviors. These perceptions align with contemporary leadership scholarship, which emphasizes that effective leadership in complex healthcare environments increasingly depends on relationships, shared meaning, and interpersonal influence rather than positional authority [5, 7].

Participants described RL as foundational to effective leadership in PHC settings, particularly because it prioritizes meaningful relationships with staff, patients, and families. This interpretation is consistent with literature describing RL as a leadership process grounded in connection, ethical engagement, and co‐constructed understanding [1, 6]. Leaders in this study perceived those relational behaviors such as intentional communication, active listening, and relational presence helped create psychologically safe environments where staff felt supported and able to contribute more openly. These perceptions echo findings from Maritsa et al. [7], who argue that RL fosters trust and cooperation, both essential in high‐pressure healthcare environments.

The perceived benefits of RL in this study included improved team cohesion, enhanced communication, and strengthened trust. Leaders felt that relational approaches encouraged staff participation, reduced interpersonal tension, and contributed to a more supportive workplace climate. These reflections align with evidence showing that RL is associated with higher organizational commitment, innovation, and job satisfaction [5]. Although participants described perceived improvements in patient interactions and service coordination, these impressions were subjective and not objectively measured. As such, the findings speak to leaders’ interpretations of RL’s potential influence on patient care rather than verified outcomes.

Despite these perceived benefits, participants also identified several challenges that complicated the practice of RL. Heavy workloads, limited resources, and entrenched hierarchical norms were described as barriers to relationship‐building and collaborative decision‐making. These contextual constraints mirror findings from Goens and Giannotti [22], who note that systemic pressures in healthcare can impede relational approaches. Nevertheless, leaders in this study expressed a sustained commitment to RL and a desire to advocate for relational practices within their organizations. This perseverance underscores the perceived value of RL in strengthening leadership capacity and supporting team resilience in under‐resourced PHC settings.

Participants also perceived a connection between RL and patient care [23], particularly through leaders’ role‐modeling of compassion, empathy, respect, and collaborative communication. This interpretation aligns with literature describing RL as supportive of high‐quality, patient‐centered care [2, 24]. However, because this study did not measure patient outcomes directly, these reflections represent leaders’ perceptions of possible care‐related benefits rather than objective evidence of improvement.

Finally, leaders identified several strategies they believed could enhance RL practice in PHC settings. These included empowering team members, seeking feedback, communicating vision clearly, and engaging in reflective practice [25, 26]. EI emerged as a particularly important competency. Evidence from Ayed [27] shows that EI strengthens nurses’ coping behaviors, emotional regulation, and clinical decision‐making skills that directly support RL. Similarly, Ramdas et al. [28] highlight that leaders who seek feedback and engage in self‐reflection improve decision‐making and alignment with team needs. A systematic review by Chaudry et al. [29] further found that emotionally intelligent healthcare leaders foster positive work environments, teamwork, and staff commitment, and may help mitigate burnout. These studies reinforce participants’ perceptions that empathy, self‐awareness, and emotional regulation are essential for practising RL effectively.

In summary, this study highlights how PHC leaders perceive and experience RL within their daily practice. The findings suggest that RL may contribute to more cohesive teams, supportive work environments, and potentially improved patient experiences, although these outcomes were not objectively measured. Integrating these perceptions with existing literature underscores the relevance of RL as a leadership approach suited to the relational, collaborative, and emotionally demanding nature of PHC settings. Strengthening relational competencies, particularly EI may support leaders in navigating contextual challenges and fostering environments that promote staff well‐being and patient‐centered care.

### 4.1. Implications for Nursing Management Practice

For nursing management, the findings suggest that RL development should be embedded in routine practice rather than limited to once‐off classroom training. Managers can use facilitated action learning groups, structured reflection, and regular feedback to strengthen self‐awareness, communication, shared decision‐making, and constructive conflict management. Senior nursing leaders should also create protected time for relationship‐building, provide equitable access to professional development, and clarify how supportive leadership can coexist with performance accountability. Because the reported benefits were participants’ perceptions, organizations implementing these approaches should evaluate them using staff, service, and patient indicators alongside qualitative feedback.

### 4.2. Limitations

In interpreting the findings of this study, several limitations should be considered. The study was conducted in two subdistricts within one health district and involved a small, purposively recruited action learning cohort; the findings are therefore context‐specific and analytically transferable rather than statistically generalizable. The initial workshop and repeated action learning sessions were not neutral data‐collection settings: they were designed to develop participants’ understanding and practice of RL and may have shaped how participants subsequently interpreted and reported it. The findings consequently reflect perceptions produced during an intervention‐informed learning process rather than pre‐existing views alone. Data were self‐reported and largely generated in a group context. Participants may have presented their leadership development favorably, and differences in status, confidence, conformity, or dominant voices may have influenced what was expressed or withheld despite facilitation and confidentiality arrangements. The researcher’s role of co‐facilitation may also have influenced participation and interpretation, although reflexive journaling, co‐coding, triangulation, peer debriefing, and member checking were used to mitigate this risk. Finally, perceived changes in team dynamics, organizational performance, patient experience, and care were not corroborated using independent staff reports, administrative data, clinical indicators, or patient‐reported measures. Future studies should include diverse sites, comparison or longitudinal designs, and multiple stakeholder and outcome measures to examine whether perceived benefits are sustained and observable beyond the action learning group. Such approaches would help reduce intervention‐related bias and strengthen the evidence base for RL in different healthcare settings.

## 5. Conclusions

This study explored PHC leaders’ perceptions of RL within an action learning process. Through reflection and dialog, participants described a gradual shift from hierarchical, authority‐driven leadership towards more relational, collaborative, and communicative practice. They perceived RL as strengthening trust, team cohesion, open communication, and psychologically safe work environments, and as supporting patient‐centered care through leaders’ role‐modeling of empathy, respect, and collaborative communication. At the same time, heavy workloads, limited resources, and entrenched hierarchical norms constrained the consistent practice of RL. Participants identified reflective practice, feedback‐seeking, clear communication of vision, and EI as key enablers. These findings suggest that RL offers a contextually relevant framework for leadership development in under‐resourced PHC settings; however, they reflect leaders’ perceptions and should not be interpreted as evidence of objectively improved organizational or patient outcomes. Nursing and health‐service managers may consider facilitated action learning, structured reflection, and EI development as components of leadership development, accompanied by evaluation using independent staff, service, and patient measures.

## Author Contributions

All authors were involved in the conception and design of the study. Lillian Kalimashe drafted the manuscript. All authors read the draft and provided input. Emmerentia du Plessis and Leepile Alfred Sehularo provided key information and contributed to the writing process. Emmerentia du Plessis and Leepile Alfred Sehularo were the study advisors and provided background information and helped with the methodology.

## Funding

This research was funded by the Gauteng Department of Health (GDoH) bursary. I have not received a number for this bursary.

## Disclosure

All authors read and approved the final manuscript.

## Conflicts of Interest

The authors declare no conflicts of interest.

## Data Availability

The data supporting this study’s findings are available upon request from the corresponding author.
